# *Pontiella agarivorans* sp. nov., a novel marine anaerobic bacterium capable of degrading macroalgal polysaccharides and fixing nitrogen

**DOI:** 10.1128/aem.00914-23

**Published:** 2024-01-24

**Authors:** Na Liu, Veronika Kivenson, Xuefeng Peng, Zhisong Cui, Thomas S. Lankiewicz, Kelsey M. Gosselin, Chance J. English, Elaina M. Blair, Michelle A. O'Malley, David L. Valentine

**Affiliations:** 1 Interdepartmental Graduate Program in Marine Science, University of California Santa Barbara, Santa Barbara, California, USA; 2 Marine Science Institute, University of California Santa Barbara, Santa Barbara, California, USA; 3 Marine Bioresource and Environment Research Center, Key Laboratory of Marine Eco-Environmental Science and Technology, First Institute of Oceanography, Ministry of Natural Resources of China, Qingdao, China; 4 Department of Chemical Engineering, University of California, Santa Barbara, California, USA; 5 Department of Ecology Evolution, and Marine Biology, University of California, Santa Barbara, California, USA; 6 Biological Engineering Program, University of California, Santa Barbara, California, USA; 7 Department of Earth Science, University of California Santa Barbara, Santa Barbara, California, USA; Georgia Institute of Technology, Atlanta, Georgia, USA

**Keywords:** polysaccharides, novel bacterium, *Kiritimatiellota*, CAZymes, sulfatases, nitrogen fixation

## Abstract

Marine macroalgae produce abundant and diverse polysaccharides, which contribute substantially to the organic matter exported to the deep ocean. Microbial degradation of these polysaccharides plays an important role in the turnover of macroalgal biomass. Various members of the *Planctomycetes*-*Verrucomicrobia*-*Chlamydia* (PVC) superphylum are degraders of polysaccharides in widespread anoxic environments. In this study, we isolated a novel anaerobic bacterial strain NLcol2^T^ from microbial mats on the surface of marine sediments offshore Santa Barbara, CA, USA. Based on 16S ribosomal RNA (rRNA) gene and phylogenomic analyses, strain NLcol2^T^ represents a novel species within the *Pontiella* genus in the *Kiritimatiellota* phylum (within the PVC superphylum). Strain NLcol2^T^ is able to utilize various monosaccharides, disaccharides, and macroalgal polysaccharides such as agar and ɩ-carrageenan. A near-complete genome also revealed an extensive metabolic capacity for anaerobic degradation of sulfated polysaccharides, as evidenced by 202 carbohydrate-active enzymes (CAZymes) and 165 sulfatases. Additionally, its ability of nitrogen fixation was confirmed by nitrogenase activity detected during growth on nitrogen-free medium, and the presence of nitrogenases (*nifDKH*) encoded in the genome. Based on the physiological and genomic analyses, this strain represents a new species of bacteria that may play an important role in the degradation of macroalgal polysaccharides and with relevance to the biogeochemical cycling of carbon, sulfur, and nitrogen in marine environments. Strain NLcol2^T^ (= DSM 113125^T^ = MCCC 1K08672^T^) is proposed to be the type strain of a novel species in the *Pontiella* genus, and the name *Pontiella agarivorans* sp. nov. is proposed.

**IMPORTANCE:**

Growth and intentional burial of marine macroalgae is being considered as a carbon dioxide reduction strategy but elicits concerns as to the fate and impacts of this macroalgal carbon in the ocean. Diverse heterotrophic microbial communities in the ocean specialize in these complex polymers such as carrageenan and fucoidan, for example, members of the *Kiritimatiellota* phylum. However, only four type strains within the phylum have been cultivated and characterized to date, and there is limited knowledge about the metabolic capabilities and functional roles of related organisms in the environment. The new isolate strain NLcol2^T^ expands the known substrate range of this phylum and further reveals the ability to fix nitrogen during anaerobic growth on macroalgal polysaccharides, thereby informing the issue of macroalgal carbon disposal.

## INTRODUCTION

Marine macroalgae are important primary producers in coastal ecosystems. They sequester about 173 TgC yr^−1^ into their biomass and are considered as part of the “blue carbon” in the ocean (1). Seaweed cultivation has been considered as one of the promising strategies to mitigate the increasing amount of anthropogenic CO_2_ and climate change (2). A recent study shows that 24% of macroalgae will eventually reach the seafloor and thus export the fixed carbon to the deep ocean (3). Polysaccharides are important components among the fixed carbon, which includes agar, carrageenan, and fucoidan (4, 5). In contrast to terrestrial plants, marine polysaccharides are usually decorated by sulfate and other functional groups, which require specialized enzymes for removal, thereby limiting the range of microbes that can access and degrade these compounds (6).

Members of the PVC superphylum (named for *Plantomycetes*, *Verrucomicrobia*, and *Chlamydiae*) include degraders of recalcitrant glycopolymers, though much of their true functional diversity has been obscured by the lack of cultivated representatives (7–10). The PVC superphylum also consists of phyla *Kiritimatiellota* and *Lentisphaerae* as well as uncultured candidate phyla from environmental samples (11). The *Kiritimatiellota* phylum was established in 2016 (previously named as *Kiritimatiellaeota*) and was recognized as the Subdivision 5 of *Verrucomicrobia* in the PVC superphylum (12, 13). The geographic distribution of 16S rRNA gene sequences reveals that bacteria in phylum *Kiritimatiellota* are common in anoxic environments ranging from the intestines of animals to hypersaline sediments and wastewater (12). However, there are only four cultivated strains reported to date, and we know little about their metabolic capabilities and functional role in the environment. The first cultivated strain, *Kiritimatiella glycovorans* L21-Fru-AB^T^, is a halophilic saccharolytic bacterium isolated from an anoxic cyanobacterial mat from a hypersaline lake on the Kiritimati Atoll (14). *Pontiella desulfatans* F1^T^ and *Pontiella sulfatireligans* F21^T^ were isolated from Black Sea sediments and are capable of degrading sulfated polysaccharides like ɩ-carrageenan and fucoidan (15, 16). *Tichowtungia aerotolerans* S-5007^T^ was isolated from surface marine sediment and can grow under microaerobic conditions (17).

In this study, we enriched and isolated a novel anaerobic bacterial strain NLcol2^T^ from the marine sediments offshore Santa Barbara, CA, USA, which belongs to the *Kiritimatiellota* phylum. We fed the strain with agar, ɩ-carrageenan, and fucoidan as carbon substrate to test whether it is able to degrade these polysaccharides or not. Among other isolates of *Kiritimatiellota*, ammonium has been identified as the nitrogen source, but nitrogen fixation has not been observed. However, macroalgal polysaccharides are depleted in nutrients including nitrogen; therefore, we used nitrogen gas as the sole nitrogen source to test its ability of nitrogen fixation. Strain NLcol2^T^ is characterized by phylogenomic, morphological, chemotaxonomic, and physiological traits. We further investigated its metabolic potential by analyzing carbohydrate-active enzymes (CAZymes), sulfatases, and nitrogenases in the genome in detail.

## MATERIALS AND METHODS

### Inoculum source, enrichment, and isolation of strain NLcol2^T^

**Fig 1:**
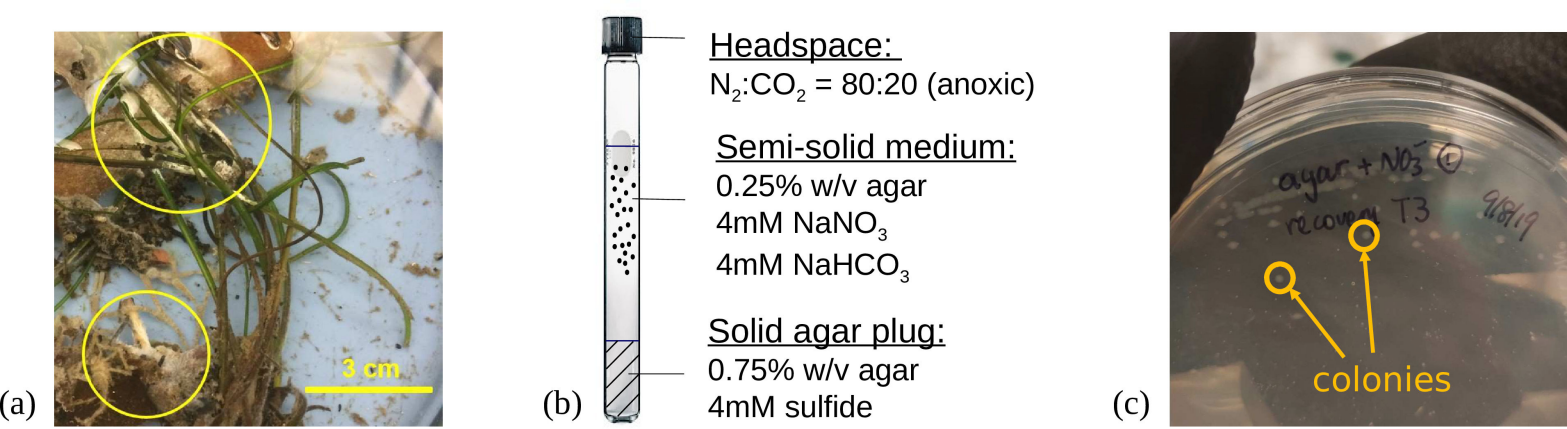
(**a**) Microbial mats collected on the surface of marine sediments were used as inoculum for the enrichment and isolation of bacterial cultures. (**b**) Anoxic enrichment culture setup with agar and sulfide gradient seawater media. (**c**) Isolation of culturable strains on agar plates. Colonies were highlighted in yellow circles.

Strain NLcol2^T^ was enriched and isolated from microbial mats found on the surface of marine sediments at Shane Seep (34.40616 N, 119.89047 W) within the Coal Oil Point seep field offshore Santa Barbara, CA, USA. Microbial mat samples were collected at 20-m depth with an *in situ* temperature of 15°C in October 2017. The seep area is characterized by a large amount of hydrocarbon gas emissions, microbial mat coverage, and high sulfide and alkalinity in sediment porewater (18–20). The samples used for inoculum contained both microbial mats and partially decomposed macroalgae (Fig. 1a). The microbial mats were scraped off their attached surface as the inoculum source. The cultures were enriched anaerobically in semisolid agar (0.25% wt/vol, BD Difco Agar, granulated) in the top layer of the sulfide gradient media (Fig. 1b) modified from Kamp et al. (2006). Cultures were maintained at room temperature in the dark and were transferred into fresh media every two to three weeks for a year.

Further isolation of strain NLcol2^T^ was performed by streaking on agar plates in an anaerobic chamber (Coy Laboratory Products; Fig. 1c). The medium is the same as the top agar medium in enrichment cultures, except that 1.5% w/v agar (BD Difco Agar, Noble) was added as both gelling agent and substrate and 2-mM sulfide added as reducing agent. The Petri dishes were kept in the anaerobic chamber at room temperature (22°C). Single colonies formed after 3 weeks and were picked from agar plates. Streak plating was repeated for three more rounds to ensure the purity of the culture. Pure culture was subsequently maintained in liquid media with D-galactose (1 g/L) as substrate at 22°C and was transferred every other week. A fully modified medium contained the following in 1,000-mL distilled water: 28.0-g NaCl, 10.0-g MgCl_2_ · 6 H_2_O, 3.8-g MgSO_4_ · 7 H_2_O, 0.6-g CaCl_2_ · 2 H_2_O, 1.0-g KCl, 37-mg K_2_HPO_4_, 4-mg Na_2_MoO_4_, 50-mg Na_2_S_2_O_5_, 2-mg FeCl_3_ · 6 H_2_O, 10.0-mL modified Wolin’s Mineral Solution (see DSMZ medium 141), 0.5-mL Na-resazurin solution (0.1% w/v), 1.0-g D-galactose, 1.0-g NH_4_Cl (optional), 0.75-g Na_2_CO_3_, 0.5-g Na_2_S · 9 H_2_O, and 10.0-mL modified Wolin’s vitamin solution (see DSMZ medium 141). All ingredients except carbonate, sulfide, and vitamins were dissolved under N_2_/CO_2_ (80:20) atmosphere in Hungate tubes or serum bottles and autoclaved. Carbonate was added from a sterile anoxic stock solution prepared under N_2_/CO_2_ (80:20) atmosphere. Sulfide and vitamins were added from sterile anoxic stock solutions prepared under 100% N_2_ gas. The purity of the isolate was checked by full-length 16S rRNA gene sequencing and observation of morphology under the microscope.

### Phylogenetic reconstruction by 16S rRNA gene

Full-length 16S rRNA gene of strain NLcol2^T^ was sequenced by GENEWIZ (Azenta Life Sciences), from colonies grown on agar plates. 16S rRNA gene sequence was searched using the website tool BLASTn (21) against the 16S rRNA database and compared to the sequence identity of the other four isolated strains in the *Kiritimatiellota* phylum.

To construct a phylogenetic tree based on the 16S rRNA gene, 106 sequences over 1,200-base pair (bp) from the *Kiritimatiellales* order in SILVA Ref NR SSU r138.1 database (released August 2020, accessed November 2021; 22) were selected for alignment. The full-length 16S rRNA genes of strain NLcol2^T^, *Tichowtungia aerotolerans* strain S-5007^T^, and two *Verrucomicrobia* (ABEA03000104, AF075271 as outgroups) were also added to the alignment using SINA Aligner v1.2.11 (23). The alignment was trimmed using the “gappyout” method in TrimAl v1.4 (24) to remove ambiguous ends and columns with >95% gaps. All trimmed nucleotide sequences represent >50% of the 1,568 alignment columns. A maximum-likelihood (ML) tree was constructed using RAxML v.8.2.9 (25) with the GTRGAMMA model of evolution. A rapid bootstrap search was stopped after 1,000 replicates with the extended majority-rule (MRE) consensus tree criterion. The best-scoring ML tree with support values was visualized in the iTOL server (26).

### Genome sequencing and analyses

Genomic DNA was extracted from the isolate cultures using FastDNA Spin Kit for Soil (MP Biomedicals, OH). Genomic DNA library preparation and sequencing were performed at the University of California Davis Genome Center on the Illumina HiSeq 4000 platform with 150-bp paired-end reads. Trimmomatic v.0.36 (27) and Sickle v.1.33 (28) were used to remove the adapter and low-quality or short reads. Trimmed reads were assembled into contigs using MEGAHIT v.1.1.1 (29). Contigs longer than 2,500 bp were kept, and the trimmed reads were mapped back to those contigs using Bowtie2 v.2.3.4.1 (30) and Samtools v.1.7 (31). Contigs were visualized using the Anvi’o v.3 interactive interface (32), and manual binning was performed based on coverage, DNA G+C content, and tetranucleotide frequency signatures. Completion and redundancy of the reconstructed genome were determined using CheckM v.1.0.7 (33).

Open reading frame (ORF) features and protein-coding gene sequences were predicted using Prodigal v.2.6.3 (34). Annotation was assigned to proteins using HMMER v.3.1b2 (35) hmmscan searching against the Pfam v.32.0 (36) and TIGRFAMs v.15.0 (37) databases with a maximum e-value of 1 × 10^−7^, corresponding to a bit score of >30 to balance the tradeoffs between false positives and missed matches. Information on protein family, domain, and conserved site were confirmed using InterProScan5 (38). The amino acid sequences of protein-coding genes were further searched against NCBI’s Conserved Domain Database (CDD; 39) using the RPS-BLAST program v.2.7.1. The cdd2cog script (40) was used to assign cluster of orthologous group (COG) categories (41) to each protein-coding gene. Protein sequences were also submitted to the BlastKOALA server (42) for KEGG Orthology (KO) ID assignments. Ribosomal RNA (rRNA) genes were determined by RNAmmer v.1.2 (43). Transfer RNA (tRNA) genes were predicted by the tRNAscan-SE 2.0 server (44). Metabolic pathways were reconstructed using the KEGG Mapper (45) and MetaCyc database (46).

For phylogenomic analyses, high-quality genomes in the *Kiritimatiellales* order from NCBI’s GenBank database and the Genome Taxonomy Database (GTDB) r95 were selected (accessed on 1 February 2021). *Opitutus terrae* PB90-1 from the *Verrucomicrobia* phylum was selected as the outgroup. All genomes meet the GTDB quality criterion based on completeness and redundancy from CheckM: completeness – 5 × redundancy > 50. A total of 120 single-copy genes were searched and aligned using GTDB-Tk v1.4.0 (47). The concatenated alignment was further trimmed using TrimAl v1.4 (24) with “gappyout” parameter, which resulted in a final alignment with 4,488 amino acid columns. A maximum-likelihood phylogenetic tree was calculated using RAxML v.8.2.9 (25) with the PROTGAMMALG model of evolution. A rapid bootstrap search was stopped after 350 replicates with MRE-based criterion. The best-scoring ML tree with support values was visualized in the iTOL server (26). The average nucleotide identity (ANI) and average amino acid identity (AAI) between genomes were calculated using the ANI/AAI calculator (48).

Carbohydrate-active enzymes were predicted using dbCAN2 meta server (49). In brief, uploaded protein sequences were searched against the dbCAN CAZyme domain HMM database v.7, CAZy database (www.cazy.org), and PPR library using HMMER, DIAMOND, and Hotpep programs, respectively (49). Only genes predicted by no less than two programs were defined as CAZymes for further analysis. To classify sulfatases into families and subfamilies, gene sequences with an annotated sulfatase domain (PF00884) were searched and classified by the SulfAtlas database v.1.1 (50) using the BLASTp program (21). Additionally, SignalP v.5 (51) was used to predict signal peptides for the translocation of sulfatases into the periplasmic space and outside of the cells.

To better understand the evolution of nitrogen fixation in the *Kiritimatiellota* phylum, reannotation and phylogenetic analysis of the *nifH* gene were performed for all 52 genomes in this phylum from NCBI’s GenBank database (accessed on 3 March 2020). The same annotation pipeline described above was used to keep consistency and allow better comparison. *nifH* gene sequences were aligned with 879 full-length *nifH* genes from the genomes of cultivated diazotrophs (https://wwwzehr.pmc.ucsc.edu/Genome879/) using MUSCLE v.3.8 (52). Two light-independent protochlorophyllide reductases were included as outgroups: ChlL from *Trichormus variabilis* ATCC 29413 (WP_011320185.1) and BchL from *Chlorobium limicola* DSM 245 (WP_012467085.1). The alignment was trimmed in Jalview v.2.10.5 (53) to remove ambiguous ends and the columns with >95% gaps. All trimmed amino acid sequences represent >81% of the alignment columns. A maximum-likelihood tree was constructed using RAxML v.8.2.9 (25) with the PROTGAMMALG model of evolution. A rapid bootstrap search was stopped after 350 replicates with MRE-based criterion. The best-scoring ML tree with support values was visualized in the iTOL server (26).

### Microscopy

To obtain high-resolution images, cell morphology was examined under the transmission electron microscope (TEM). For TEM imaging, cells grown on the agar plates were fixed with modified Karnovsky’s fixative (2% paraformaldehyde and 2.5% glutaraldehyde in 0.1-M sodium phosphate buffer) and spun down into a cell pellet. Cells were rinsed in 0.1-M sodium phosphate buffer and fixed again with 1% osmium tetroxide in the same buffer. After another rinse, they were dehydrated in 50% EtOH, 75% EtOH, 95% EtOH, 100% EtOH, and propylene oxide twice. Cells were pre-infiltrated in 1:1 propylene oxide:resin (Epon/Araldite mixture) overnight, infiltrated in 100% resin, and embedded in fresh resin at 60°C overnight. Ultrathin sections were cut using a Diatome diamond knife. Sections were picked up on copper grids and imaged in a FEI Talos 120C transmission electron microscope at the Biological Electron Microscopy Facility, University of California Davis.

### Chemotaxonomic analysis

The cellular fatty acid composition of strain NLcol2^T^ was determined from cells grown at 22°C to late-log phase in a liquid medium with 1.0-g/L D-galactose as carbon source and nitrogen gas as nitrogen source. Cells were centrifuged down at 10,000 × *g* for 10 min and were frozen at −80°C.

Cellular fatty acids were extracted twice using a modified Folch method (54) with a chloroform/methanol mixture (2:1) and tridecanoic acid as an internal standard. The samples were partitioned, and the organic phase containing the total lipid extract (TLE) was retained. Transesterification of the TLE was performed by adding toluene and 1% sulfuric acid in methanol to the TLE after it was brought to complete dryness under N_2_. The acidic methanol/toluene TLE was heated at 90°C for 90 min to produce fatty acid methyl esters (FAMEs). The FAMEs were extracted from the acidic methanol by adding hexane and water, vortexing, centrifuging, and removing the top (hexane) fraction to a new vial twice. The combined transesterified hexane extracts were dried under N_2_ to a final volume of 300 µL. Each extract was spiked with methyl heptadecanoate to calculate the recovery of the internal standard and analyzed by gas chromatography with flame ionization detection (GC-FID).

Concentration analysis was performed with an HP 5890 Series II GC-FID. Chromatography was performed with a 30-m × 0.25-mm internal diameter (ID), 0.25-µm pore size, fused silica Omegawax capillary column with a splitless 1-µL injection. The initial oven temperature was set at 50°C and held for 2 min, followed by a 10°C min^−1^ ramp to 150°C, then a 5°C min^−1^ ramp to the final temperature of 265°C. A certified reference material (FAME 37, Supelco) was run to calculate retention times and identify peaks. Peak identification was further confirmed by their mass spectra.

Analyses of catalase, oxidase, and API ZYM assay for semiquantitation of enzymatic activities (e.g., β-galactosidase) were carried out by DSMZ Services, Leibniz-Institut DSMZ—Deutsche Sammlung von Mikroorganismen und Zellkulturen GmbH, Braunschweig, Germany.

### Physiology

The bacterial growth of strain NLcol2^T^ was monitored by measuring the optical density (OD) of liquid cultures at 600-nm wavelength. Growth at different temperature (4, 10, 14, 22, 26, 31, 37, 55°C), salinity (0%, 1%, 2%, 2.5%, 3%, 4%, 5%, 6% NaCl), and pH (4.0, 5.0, 5.5, 6.0, 6.5, 7.0, 8.0, 9.0) conditions were determined in triplicates when growing on D-galactose with ammonium supplied. Growth was tested on various substrates (1 g/L) in triplicates at optimum temperature, salinity, and pH conditions with ammonium supplied: D-glucose, D-galactose, D-fructose, L-fucose, L-rhamnose, D-mannose, D-mannitol, meso-inositol, D-arabinose, D-xylose, D-cellobiose, lactose, sucrose, maltose, xylan from corn core (TCI), starch (Sigma-Aldrich), cellulose (Sigma-Aldrich), alginic acid (Acros Organics), agarose (Sigma-Aldrich), agar (BD Difco Agar, Noble), ɩ-carrageenan (TCI), fucoidan from *Macrocystis pyrifera* (Sigma-Aldrich), commercially bought dried red algae (*Porphyra* spp.), commercially bought dried brown algae (*Saccharina japonica*), and the giant kelp (*Macrocystis pyrifera*) harvested from offshore Santa Barbara, CA, USA.

To test the utilization of several nitrogen sources by strain NLcol2^T^, we cultured them with sodium nitrate (1 g/L) and ammonium chloride (1 g/L) and without any nitrogen species supplemented in the liquid media. Two sets of tubes with headspace gases of nitrogen gas or helium gas were made as experimental and control groups, respectively. Triplicate cultures were supplied with 1-g/L D-galactose as substrate and incubated at room temperature (22°C) for 14 days. Growth was monitored by OD (600 nm) measurements.

### Metabolite analysis from galactose fermentation

To quantify the metabolic products of strain NLcol2^T^ from galactose fermentation, cultures were grown in triplicates at room temperature (22°C) with D-galactose as the carbon source for 10 days. No ammonium was added to the media, and N_2_ gas served as the sole nitrogen source. Growth was monitored by measuring OD at 600-nm wavelength. In addition, 2 mL of culture was subsampled each day (twice a day during the exponential phase) for the quantification of metabolites.

The chromatography protocols used in this study are similar to those previously described (55, 56). Galactose, acetate, succinate, and malate concentrations were measured on an Agilent Infinity 1260 (Agilent Technologies, Santa Clara, CA, USA) high-performance liquid chromatograph (HPLC) using an Aminex HPX-87H analytical column (part no. 1250140, Bio-Rad, Hercules, CA, USA) protected by, first, a 0.22-µm physical filter, followed by a Coregel USP L-17 guard cartridge (Concise Separations, San Jose, CA, USA). Separations were performed at 60°C with a flow rate of 0.6-mL/min and a 5-mM sulfuric acid (H_2_SO_4_) mobile phase. Acetate, succinate, and malate were measured using a variable wavelength detector set to 210 nm, while galactose was measured using a refractive index detector set to 35°C. Samples and standards for HPLC were acidified to a concentration of 5-mM H_2_SO_4_, incubated for 5 min at room temperature, and spun at maximum speed in a tabletop centrifuge for 5 min to pellet bacterial cells. The samples were removed from above the cell pellet, and 0.22-µm filtered through a polyethersulfone (PES) membrane into HPLC vials with 300-µL polypropylene inserts. Standard curves for each compound of interest were constructed using triplicate standards of 0.1, 0.5, and 1.0 g/L. Peaks were integrated using OpenLab CDS analysis software (version 2.6, Agilent Technologies).

Hydrogen gas production was measured on a Fisher Scientific TRACE 1300 gas chromatograph (Thermo Fisher Scientific, Waltham, MA, USA) using a TRACE TR-5 GC Column (part no. 260E113P, Thermo Fisher Scientific) at 30°C, with an Instant Connect Pulsed Discharge Detector (PDD; part no. 19070014, Thermo Fisher Scientific) at 150°C, and ultrahigh purity He as a carrier gas. All injections of samples and standards were 100 µL in volume. Supplier-mixed standards of 50 ppm, 500 ppm, and 1% hydrogen were run before and after injecting samples, and hydrogen peaks were integrated using Chromeleon Chromatography Data System (CDS) Software (version 6.8, Thermo Fisher Scientific). CO_2_ was not considered due to the carbonate-buffered medium and N_2_/CO_2_ atmosphere.

A tentative fermentation balance was formulated based on the concentrations of galactose, succinate, acetate, malate, and hydrogen measured above. The changes in concentrations in mmol/L were taken as coefficients for these compounds.

### Metabolite analysis from agar and ɩ-carrageenan degradation

Cultures were grown at 33°C with 1-g/L agar (BD Difco Agar, Noble) and 1-g/L ɩ-carrageenan (TCI) as carbon sources and ammonium was supplied in the media. In addition, 10 mL of culture was sampled and filtered through 0.22-μm PES membrane (Millipore Millex) both on Day 0 immediately after inoculation, as well as on Day 9 and Day 7 for agar and carrageenan incubations, respectively. Growth was confirmed by OD (600 nm) measurements.

Agar and carrageenan concentrations were quantified as polymeric galactose, the main sugar component of the two polymers. Polymeric galactose was quantified as the difference between total galactose and free galactose. To measure total galactose, 5 mL of 0.22-μm-filtered media was acid hydrolyzed to cleave glycosidic linkages and release galactose. Samples were hydrolyzed in 1-M HCl at 100°C for 20 h. Following hydrolysis, samples were neutralized by N_2_ evaporation and diluted 1:1,000 with ultrapure water. Galactose was quantified using high-performance anion exchange chromatography with pulsed amperometric detection (HPAEC-PAD) on a DIONEX ICS5000+ equipped with a CarboPac PA10 column using an isocratic elution of 18-mM NaOH for 20 min (57). Free galactose was measured by HPAEC-PAD before acid hydrolysis. Incubation media was 1:25 diluted with ultrapure water to reduce the salt concentration and quantified using the same gradient program described above.

Acetate and succinate concentrations were measured on the Agilent Infinity 1260 HPLC using a similar protocol described above in the “Metabolite analysis from galactose fermentation” section, except using a refractive index detector.

### Acetylene reduction assay

To test the nitrogenase activity of strain NLcol2^T^ when growing with nitrogen gas as the sole nitrogen source, an acetylene reduction assay was performed following Hardy et al. (1968). In short, acetylene (C_2_H_2_) can be reduced to ethylene (C_2_H_4_) when nitrogenases actively fix nitrogen gas at the same time. Cultures were grown on D-galactose in triplicates at 22°C, and triplicate media bottles without inoculation were used as controls. In addition, 1.2 mL of acetylene was injected into all culture and control bottles, which contained 80 mL of liquid and 80 mL of headspace pressurized at 150 kPa at the beginning. Gas concentrations and OD_600_ were measured at six time points during the 18-day incubation. Acetylene and ethylene concentrations were resolved on a Shimadzu 8A gas chromatograph with a flame ionization detector (GC-FID). Furthermore, 1.5-mL samples and standards were injected and then carried by N_2_ at a flow rate of 20 mL/min through an n-octane on Res-Sil C packed column (Restek, Centre County, PA, USA) set at 25°C. Moreover, 0.5% and 1.0% GASCO calibration gas mixtures of acetylene and ethylene (Cal Gas Direct Incorporated, Huntington Beach, CA, USA) were used for the standard curves.

## RESULTS AND DISCUSSION

### Phylogenetic analyses

Phylogenetic placement of strain NLcol2^T^ was determined by comparing full-length 16S rRNA gene, single-copy genes, and whole-genome similarity metrics including ANI and AAI.

Strain NLcol2^T^ was classified within the R76-B128 clade (*Pontiellaceae* family in GTDB database) of the *Verrucomicrobia* phylum under the current SILVA taxonomy (SILVA Ref NR SSU r138.1). Full-length 16S rRNA gene of the isolate shares 84.1%, 88.9%, 92.9%, and 94.5% identity with the four reported cultivated strains in the *Kiritimatiellota* phylum: *Kiritimatiella glycovorans* strain L21-Fru-AB^T^, *Tichowtungia aerotolerans* strain S-5007^T^, *Pontiella sulfatireligans* strain F21^T^, and *Pontiella desulfatans* strain F1^T^, respectively. Strain NLcol2^T^ is more closely related to *P. desulfatans* and *P. sulfatireligans* than *K. glycovorans* and *T. aerotolerans*. The 16S rRNA gene identities compared to *P. desulfatans* and *P. sulfatireligans* were absolutely higher than the 86.5% threshold for family level but fall on the edge of the threshold for a new genus as 94.5% (58). A maximum-likelihood tree of 16S rRNA gene sequences from the *Kiritimatiellota* phylum was reconstructed by RAxML (Fig. S1). The R76-B128 clade (*Pontiellaceae* family) formed a monophyletic group with the MSBL3 clade (*Tichowtungiaceae* family) as the sister group, both of which are in a different cluster from the *Kiritimatiellaceae* family. It is clear that strain NLcol2^T^ is not affiliated with *K. glycovorans* within the *Kiritimatiellaceae* family nor with *T. aerotolerans* within the *Tichowtungiaceae* family (MSBL3 clade) but belongs to the *Pontiellaceae* family (R76-B128 clade) within the *Kiritimatiellales* order as do *P. desulfatans* and *P. sulfatireligans* (15).

**Fig 2:**
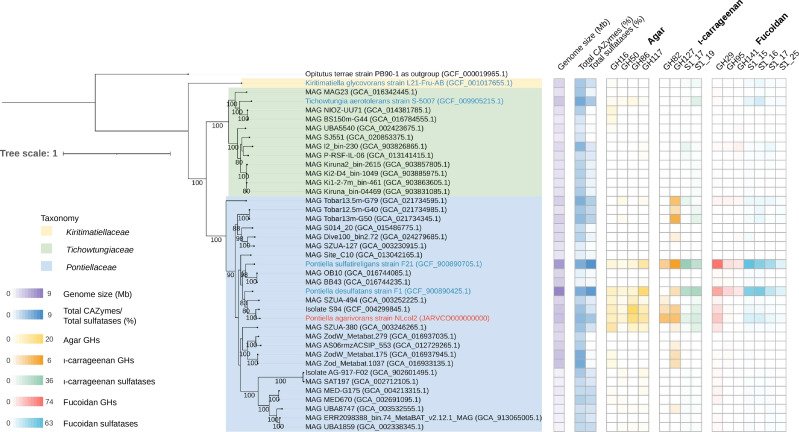
Concatenated maximum-likelihood phylogenomic tree of 120 bacterial single-copy genes from 39 genomes in the *Kiritimatiellales* order. Strain NLcol2^T^ is labeled in red, and other cultivated strains are labeled in blue. A *Verrucomicrobia* genome was selected as an outgroup, and the tree was rooted there. Bootstrap values over 80 are shown on the nodes. Genome size (Mb), total CAZymes (%), and total sulfatases (%) as a percentage of all protein-coding genes in each genome are presented as reference. The number of glycoside hydrolase (GH) and sulfatase homologs involved in the degradation pathways of agar, ɩ-carrageenan, and fucoidan is presented in the heatmap.

To resolve the phylogeny of strain NLcol2^T^ in detail, we further performed genome-level phylogenetic analyses using the Genome Taxonomy Database Toolkit (47). A concatenated phylogenomic tree was reconstructed from 120 bacterial single-copy genes of genomes in the *Kiritimatiellales* order (Fig. 2). Here, strain NLcol2^T^ falls within the *Pontiellaceae* family with a bootstrap value of 100. Additionally, the AAI values of the genomes between strain NLcol2^T^ and *P. desulfatans* and *P. sulfatireligans* are 69.94% and 68.51%, which are slightly above the threshold of 65% for the same genus (59). However, within *Pontiella* genus, it represents a different group from *P. desulfatans* and *P. sulfatireligans*. Moreover, the ANI values of the genomes between strain NLcol2^T^ and *P. sulfatireligans* and *P. desulfatans* are 72.73% and 73.71% respectively, which was much lower than the 95% ANI criterion for the same species (60, 61). Therefore, we propose that strain NLcol2^T^ represents a novel species within the *Pontiella* genus according to the phylogenetic analyses above.

### General features of the genome

The draft genome of strain NLcol2^T^ is 95% complete with 4% redundancy. The genome consists of 12 contigs (N50 is 1,265,434 bp) with a total length of 4,436,865 bp, and the mean coverage is 593×. DNA G + C content is 52.4 mol%. 5S, 16S, and 23S rRNA genes and 50 tRNA genes were found in the genome.

A total of 3,611 open reading frame (ORF) features were predicted by Prodigal, among which 2,757 proteins in the genome were assigned with COG functional category codes. The number of genes in each functional category is shown in Figure S2. More genes are involved in carbohydrate (*n* = 260) and amino acid (*n* = 188) transport and metabolism than those of nucleotides (*n* = 62) and lipids (*n* = 61), which is similar to that in *Kiritimatiella glycovorans* (12). A further detailed analysis of genes involved in macroalgal polysaccharide degradation and nitrogen fixation is presented in the “Anaerobic degradation of macroalgal polysaccharides” and “Nitrogen fixation” sections below.

### Morphologic and chemotaxonomic characterization of strain NLcol2^T^

**Fig 3:**
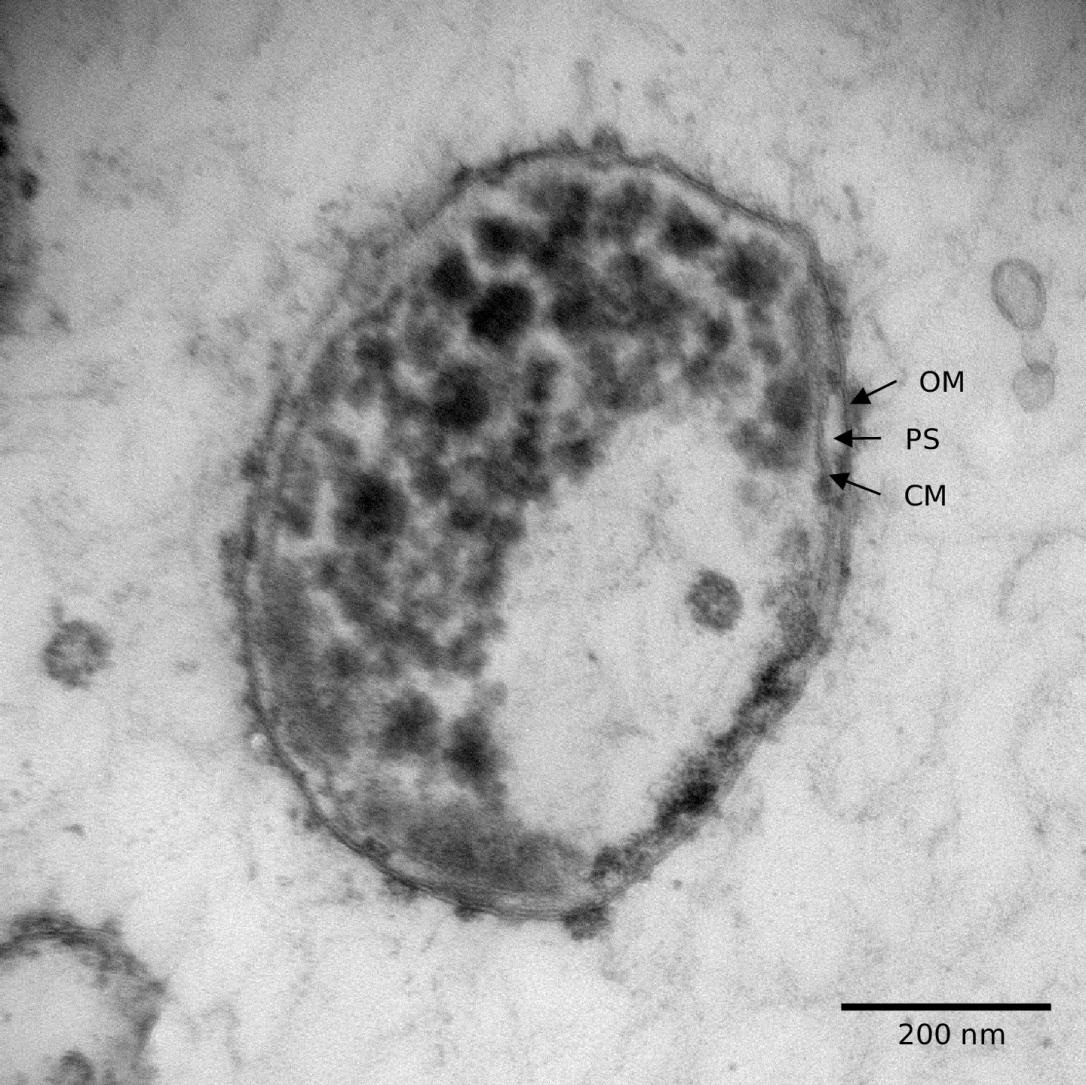
Transmission electron microscopy (TEM) image of strain NLcol2^T^ shows the cell of ~1 μm in diameter with a Gram-negative cell wall structure. OM, outer membrane; PS, periplasmic space; CM, cell membrane.

Single colonies on agar plates were white or ivory, circular, and smooth after growing anaerobically for 2 weeks at 22°C. Bacterial cells of strain NLcol2^T^ have a round to ovoid shape with a size of 1 µm in diameter observed under the microscope (Fig. 3). Cells divided by binary fission and genes of bacterial cell division complex including the FtsZ family were present. No motility or flagella were observed, although a full set of genes coding for flagellar assembly was present in the genome. No spore formation was observed. A Gram-negative cell wall structure of the outer membrane, periplasmic space, and cytoplasmic membrane was shown by electron microscopy (Fig. 3). There are also genes coding for proteins involved in lipopolysaccharide export and peptidoglycan synthesis in the genome. Some bacteria in the PVC superphylum exhibit compartments inside the cells (62), but like other strains in the *Kiritimatiellota* phylum, no compartmentalization of the cytoplasm was observed in strain NLcol2^T^. There were unknown inclusions or granules present inside the cells, and genes involved in the synthesis and utilization of polyphosphate and glycogen were found in the genome, which may serve as phosphate and energy storage materials, respectively.

Major cellular fatty acids (>10% of total) of strain NLcol2^T^ include C18:0, *i-*C12:0, *i-*C18:0, and *i*-C14:0, in order of abundance. The major cellular fatty acid profile is quite different from *K. glycovorans* and *T. aerotolerans* but almost the same as that in *P. desulfatans* and *P. sulfatireligans*, except that *P. sulfatireligans* also has *i*-C16:0 as one of the major components (Table 1). Again, this agrees with the phylogenetic placement of strain NLcol2^T^ in the *Pontiella* genus, being more closely related to *P. desulfatans* than *P. sulfatireligans*. However, strain NLcol2^T^ can be further distinguished by a relatively higher composition of *i*-C18:0 than *i*-C14:0, while *P. desulfatans* has more *i*-C14:0 than *i*-C18:0 (Table S1). Other cellular fatty acids detected in strain NLcol2^T^ include C16:0, *i-*C16:0, C20:0, and *i-*C20:0 (Table S1).

**TABLE 1:** Comparison of phenotypic characteristics between strain NLcol2^T^ and four other isolated strains in the *Kiritimatiellota* phylum^*a*^

Strains	*P. agarivorans* NLcol2^T^	*P. desulfatans* F1^T a)^	*P. sulfatireligans* F21^T a)^	*K. glycovorans* L21-Fru-AB^T b)^	*T. aerotolerans* S-5007^T c)^
Isolation source	Microbial mat on marine sediment	Anoxic marine sediment	Anoxic marine sediment	Hypersaline microbial mat	Marine sediment
Cell shape	Spherical	Spherical	Spherical	Spherical	Spherical
Cell size (μm)	1.0	0.5–1.2	0.5–1.0	1.0–2.0	0.5–0.8
Motility	-	-	-	-	-
Genome size (Mbp)	4.44	8.66	7.40	2.95	3.88
DNA G + C content (mol%)	52.4	56.0	54.6	63.3	53.1
Major cellular fatty acids (>10% of total)	C18:0, *i-*C12:0, *i-*C18:0	C18:0, *i-*C12:0, *i-*C14:0	C18:0, *i-*C12:0, *i-*C18:0	*i-*C14:0, C18:0	C18 : 0, *i-*C12 : 0, *i-*C18 : 0, *i-*C16 : 0
Catalase activity	-	-	-	-	Weak
Oxidase activity	-	-	+	-	-
Growth temperature (°C)					
Range	10–37	10–30	0–25	20–40	15–45
Optimum	31	25	25	28	33–35
Growth salinity (g/L NaCl)					
Range	10–60	10–31	10–50	20–180	5–80
Optimum	25–30	23	23	60–70	30–40
Growth pH					
Range	6.0–9.0	6.5–8.5	6.0–8.5	6.5–8.0	6.0–8.5
Optimum	8.0	7.5	7.5	7.5	7.0–7.5
Substrate utilization					
Glucose	+	+	+	+	+
Galactose	+	+	+	+/-	+
Fructose	+	+	+	-	+
Fucose	-	+	+	-	NA
Rhamnose	-	+	+	+/-	+
Mannose	+	-	+	+	-
Mannitol	+	-	+	-	-
Arabinose	-	+	-	-	+
Xylose	+	+	+	+	-
Lactose	+	+	+	-	NA
Cellobiose	+	+	+	-	+
Sucrose	+	+	+	-	-
Maltose	+	+	+	-	-
Fucoidan	-	+	+	+/-	NA
ɩ-Carrageenan	+	-	+	+/-	NA
Xylan	+	-	-	NA	NA
Agar	+	-	-	-	-
Major nongaseous fermentation products*	Succinate, acetate, malate	Acetate, ethanol, lactate	Acetate, ethanol, lactate	Ethanol, acetate	Acid (maybe acetate**)
Nitrogen sources	N_2_, NH_4_^+^	NH_4_^+^	NH_4_^+^	NH_4_^+^	NH_4_^+^

^*a*^ Notations: NA, data not available. +, positive; -, negative; +/-, unstable, ceasing growth upon the second transfer. Data for strains other than NLcol2^T^ were referenced from literatures: (a) (16); (b) (12); (c) (17). *Substrates were D-galactose for strain NLcol2^T^ and D-glucose for other strains. **For strain S-5007^T^, acetate production was predicted from genomic data.

Strain NLcol2^T^ tested negative for both catalase and oxidase, which is common in strict anaerobes (Table 1). β-galactosidase was tested positive with ~5 nanomoles of substrate hydrolyzed in the API Zym assay.

### Physiology of growth

Strain NLcol2^T^ exhibited consistent growth between 10-37°C (optimum 31°C), with NaCl concentration between 10 and 60 g/L (optimum 25–30 g/L) and with pH 6.0–9.0 (optimum pH 8.0) when D-galactose was utilized as the substrate. It was determined as a mesophilic and neutrophilic bacterium, which is similar to the other four isolated strains from the *Kiritimatiellota* phylum (Table 1). Growth with ammonium supplied in the medium was faster than when dependent on nitrogen fixation. The doubling times were 15 h and 65 h when growing with and without ammonium, respectively, at room temperature (22°C). Strain NLcol2^T^ was considered as obligately anaerobic, being unable to grow with the presence of oxygen and even in a non-reduced medium lacking sulfide as the reducing agent.

Strain NLcol2^T^ was able to grow on various carbohydrate substrates under optimal conditions with ammonium supplied, which includes D-glucose, D-galactose, D-fructose, D-mannose, D-mannitol, D-xylose, D-cellobiose, lactose, sucrose, maltose, xylan, agarose, agar, and ɩ-carrageenan (Fig. S3). No growth was observed when supplied with L-fucose, L-rhamnose, D-arabinose, meso-inositol, starch, cellulose, alginic acid, or fucoidan from *Macrocystis pyrifera*.

**Fig 4:**
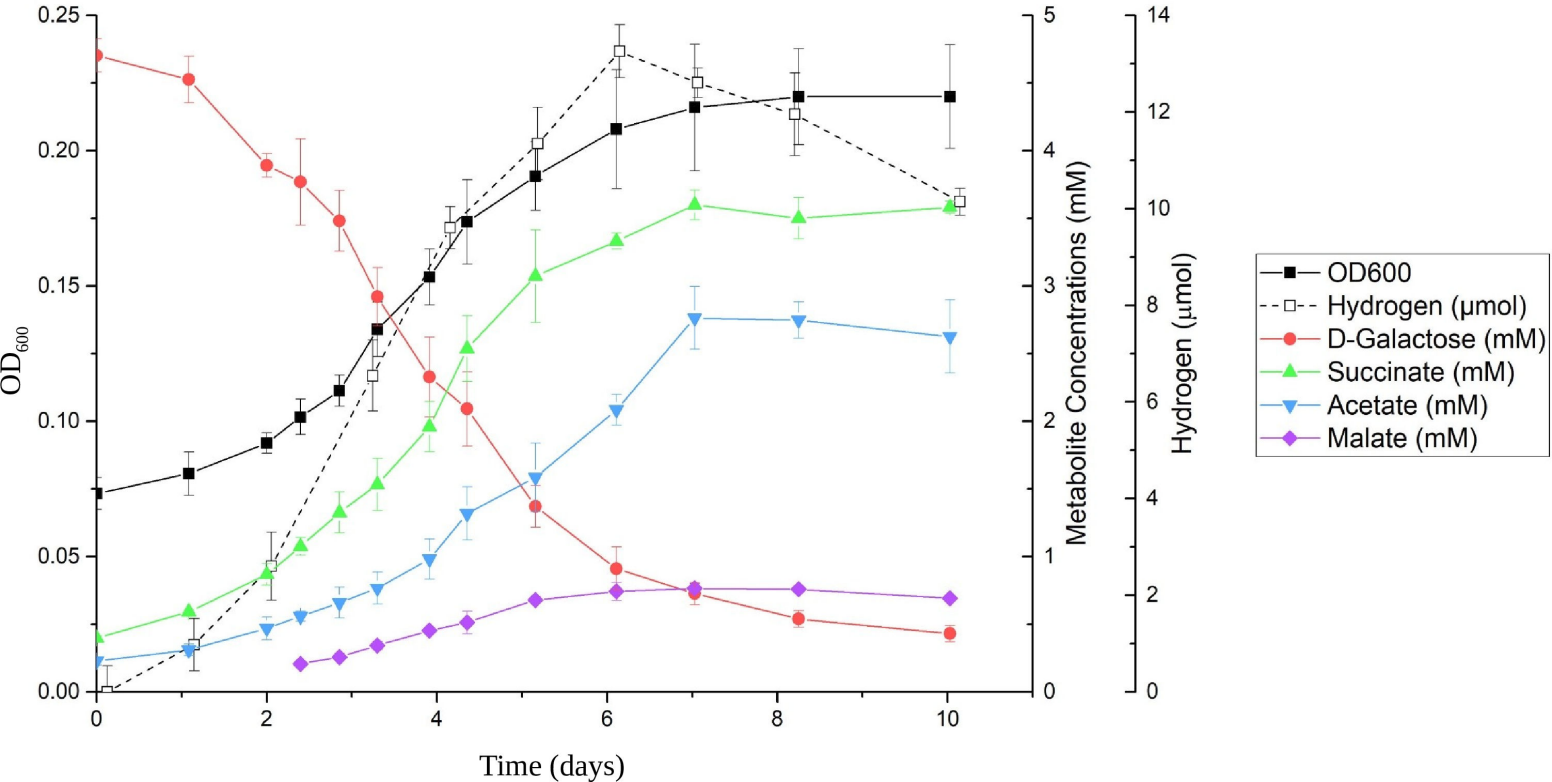
Time series of major metabolites from D-galactose fermentation by strain NLcol2^T^. The growth curve was measured by optical density at 600 nm (OD_600_). D-Galactose decreased while fermentation products of succinate, acetate, malate, and hydrogen gas were produced during anaerobic bacterial growth on D-galactose.


When growing on D-galactose, major fermentation products formed were succinate and acetate, with small amounts of malate and hydrogen gas also detected during the incubation (Fig. 4). Initially, the culture was supplied with 4.71 ± 0.12-mM D-galactose, and only 0.43 ± 0.06 mM D-galactose remained after the 10-day incubation period. Taking all fermentation products into consideration, the fractional electron recovery for galactose fermentation by strain NLcol2^T^ was about 75%. The remaining electrons could be shunted to and utilized by nitrogen fixation and biomass formation. A tentative fermentation balance was formulated as below, including measured fermentation products:


Galactose→Succinate+Acetate+Malate+Hydrogen+{biomass}

$${4.3C}_{6}H_{12}O_{6}\to {3.2C}_{4}H_{6}O_{4}+{2.4C}_{2}H_{4}O_{2}+{0.7C}_{4}H_{6}O_{5}+{0.12H}_{2}+\{biomass\}$$

### Anaerobic degradation of macroalgal polysaccharides

#### CAZyme analyses

Microbial degradation of macroalgal polysaccharides involves complex metabolic pathways and requires a large number of enzymes during the process (63–66). CAZymes, especially glycoside hydrolases (GHs) and polysaccharide lyases, can break down polysaccharides into oligosaccharides (67). In the genome of strain NLcol2^T^, 202 genes (5.6% of predicted ORFs) were predicted to be CAZymes and associated carbohydrate-binding modules (CBM) by dbCAN2 meta server (49; Table S2). Among these, 164 genes were annotated to be in the GH families. GH2, GH29, GH86, and GH117 are the most abundant families mainly represented by β-galactosidase, α-L-fucosidase, β-agarase, and α-1,3-L-neoagarooligosaccharide hydrolase. Furthermore, 100 GHs were predicted with signal peptide sequences indicating that 61% of GHs target the cell membrane or can be exported outside of the cell. Extracellular and membrane-associated GHs may hydrolyze large extracellular polymers that cannot otherwise enter the cell. Four porins and nine sugar transporters of the major facilitator superfamily were also present in the genome, which may help with the acquisition of carbohydrate molecules by the cell.

#### Sulfatase analyses

As most marine polysaccharides are sulfated, another group of enzymes called sulfatases is needed in the degradation pathway, which can cleave sulfate ester groups off the carbohydrate backbone (50). It has been shown that *Kiritimatiellota* and PVC superphylum have large numbers of copies of sulfatase genes in their genomes (15), and it is the same case in strain NLcol2^T^. We found 165 sulfatase genes (PF00884), comprising 4.6% of predicted ORFs in the genome.

Sulfatases are activated via posttranslational modification by other enzymes before functioning. The most common one is the formylglycine-generating enzyme (FGE), which transforms a cysteine or serine residue into a catalytic formylglycine (68). These fGly-sulfatases are classified as type I sulfatases (family S1), which contain all carbohydrate sulfatases and are the largest sulfatase family (6). Sulfatases were classified into 22 subfamilies in the SulfAtlas database (50), all of which belong to family S1 fGly-sulfatases (Table S3). The most abundant subfamilies (>5% of total sulfatases) in strain NLcol2^T^ are S1_16, S1_7, S1_15, S1_24, S1_8, S1_19, S1_17, and S1_20. Homologous sulfatases with known enzymatic activities within these subfamilies include D-galactose-6-sulfate 6-O-sulfohydrolase, endo-/exo-xylose-2-sulfate-2-O-sulfohydrolase, endo-/exo-galactose-4-sulfate-4-O-sulfohydrolase, endo-3,6-anhydro-D-galactose-2-sulfate-2-O-sulfohydrolase, and exo-fucose-2-sulfate-2-O-sulfohydrolase, and the known substrates of these sulfatases include alpha-/iota-/kappa-carrageenan, fucan, and ulvan (Table S3). These results imply that strain NLcol2^T^ has the potential to target a vast variety of sulfated polysaccharides, similar to isolates *K. glycovorans* (12), *P. desulfatans*, and *P. sulfatireligans* (15, 16). However, due to the limited number of characterized fGly-sulfatases, there are still many unknowns about the specific substrates and/or reactions catalyzed by sulfatases in each subfamily (50). In addition, 128 sulfatases have the best match genes from organisms in the PVC superphylum and 32 from *Bacteroidota*, and 96% of sulfatases (n = 158) were predicted to have a signal peptide sequence, indicating most sulfatases could be membrane-anchored or exported outside of the cell.

Although less well studied, the anaerobic sulfatase-maturing enzyme can mature either cysteine or serine sulfatases under anaerobic conditions (69, 70). There are also five genes encoding formylglycine-generating enzyme and one encoding anaerobic sulfatase-maturing enzyme, which are essential for the activation of sulfatase by posttranslational modification (68, 69).

#### Growth on macroalgal polysaccharides

We further confirmed the ability of strain NLcol2^T^ to grow on different macroalgal polysaccharides in live cultures. Bacterial growth was observed in anaerobic cultures with agarose, agar, and ɩ-carrageenan, but not fucoidan. Many commercially bought algal polysaccharides are contaminated with co-extracted impurities, so we took direct measurements of polysaccharides to confirm the degradation of agar and ɩ-carrageenan by strain NLcol2^T^. Agar and ɩ-carrageenan concentrations were quantified as polymeric galactose after acid hydrolysis. Polymeric galactose of agar and ɩ-carrageenan decreased by 88% and 91%, respectively, while the fermentation products of succinate and acetate increased by 88%–93% along with bacterial growth (Fig. S4). The carbon recovery rates are 78% and 87% for agar and ɩ-carrageenan degradation, respectively. This indicates that strain NLcol2^T^ is able to degrade agar and ɩ-carrageenan and their growth was mainly fueled by these polysaccharides but not the impurities. This is the first strain reported with the ability to utilize agar as substrate in the *Kiritimatiellota* phylum. We further tested their growth on seaweeds and cells also exhibited consistent growth on dried red algae (*Porphyra* spp.) and dried brown algae (*Saccharina japonica*), but not on the giant kelp (*Macrocystis pyrifera*). Since agar, porphyran, and carrageenan are all sulfated polysaccharides extracted mainly from red algae with a similar structure (5, 71), it is not surprising that cells can grow on *Porphyra* spp. directly.

**Fig 5:**
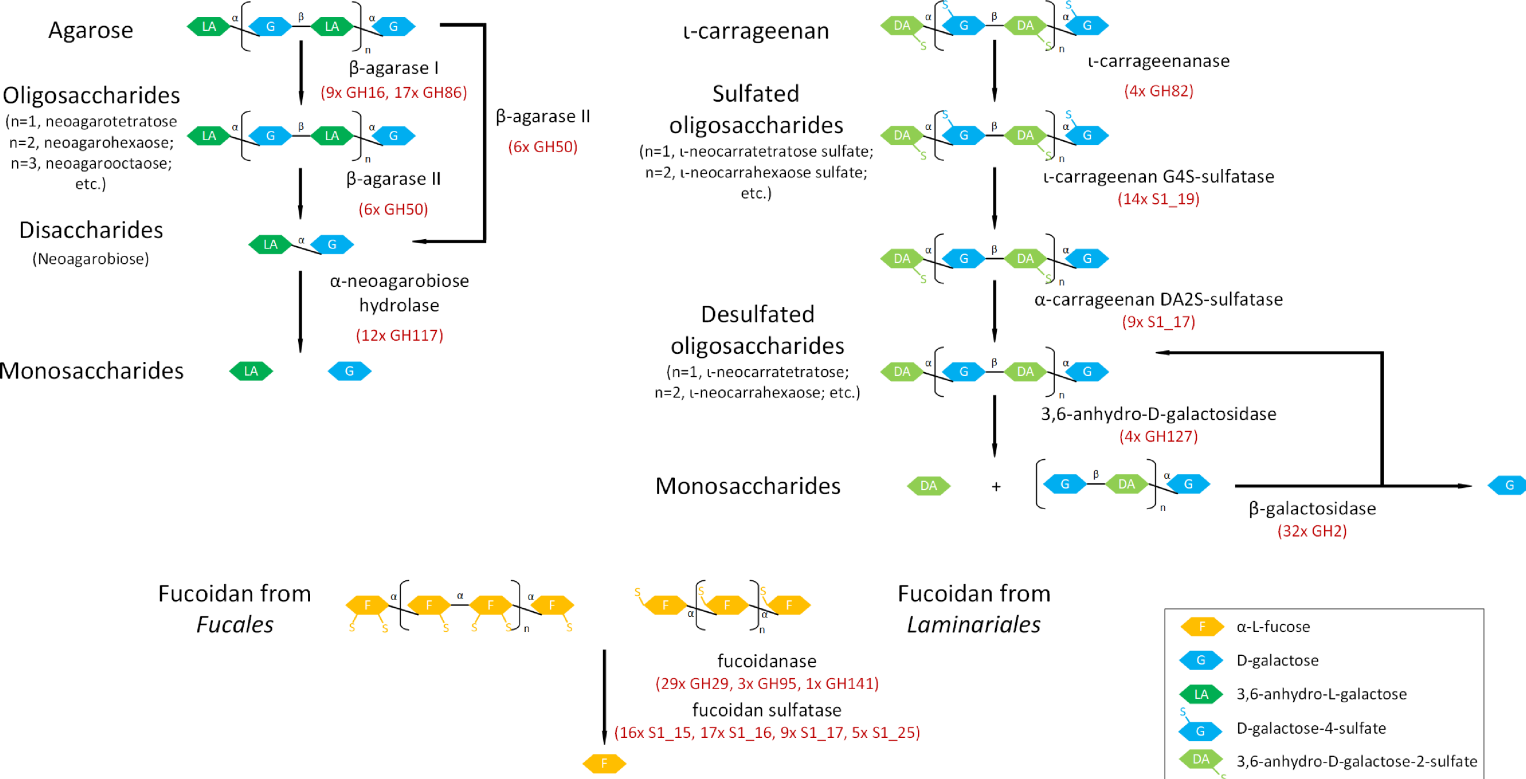
Homologs of key enzymes involved in the metabolic pathways of agar, ɩ-carrageenan, and fucoidan degradation were found in the genome. The number of gene copies encoding homologs of enzymes in the pathway is also listed. GH, glycoside hydrolase; S1_#, sulfatase classified in S1 family_subfamily. The GHs and sulfatases listed are families/subfamilies including putative agarases, carrageenans, and fucoidanases. These annotations need further subfamily-based annotation and biochemical verification.

Agar is a mixture of agarose and agaropectin, which is commonly used as a solidifying agent for culture media. Agarose is composed of alternating α-1,3-linked D-galactose and β-1,4-linked 3,6-anhydro-α-L-galactose with little sulfate modification, while agaropectin is heavily modified with sulfate (72–74). Carrageenan is structurally related to agarose, except the β-linked unit is D-galactose-6-sulfate (73). Fucoidan is also a sulfated polysaccharide composed mainly of L-fucose units adorned with sulfate esters, while minor xylose, galactose, mannose, and glucuronic acid can be present too (75). Algal polysaccharide degradation has been well studied in *Zobellia galactanivorans* Dsij^T^, the marine *Bacteroidota* model for the discovery of agarases, porphyranases, and carrageenases (64, 65, 76). We found not only potential genes involved in the degradation pathways of agar and ɩ-carrageenan but also fucoidan (Fig. 5; Table S4). Homologous genes encoding for potential β-agarases, ι-carrageenanases, and associated sulfatases were found in the genome of strain NLcol2^T^ and could be involved in degrading agar and ɩ-carrageenan into galactose and anhydrogalactose, which then can be directed to the central metabolism for energy. Potential fucoidanases were also found in the genome, but this contrasts with the experimental observation that cells did not grow with fucoidan from *M. pyrifera* as the sole carbon source. However, bacterial growth was not supported by L-fucose either (see the “Physiology of growth” section above), indicating that strain NLcol2^T^ may house potential fucoidanases only to remove fucose from fucoidan, but cannot further metabolize fucose and cannot gain energy from fucoidan degradation to support its growth. Alternatively, these genes may not encode fucoidanases to degrade fucoidan but may encode enzymes for other purposes, for example, removing the fucose cap from mucin-like molecules (77).

A neighborhood analysis of the genome shows that GHs and sulfatases are often located nearby (within the distance of five genes), suggesting certain sulfatases and glycoside hydrolases could be regulated together to degrade sulfated polysaccharides (78). In some cases, histidine kinase (PF07730, PF02518), response regulator (PF00072), and TonB-dependent transporters (PF00593, PF03544) are in the neighborhood too. The histidine kinase and response regulator together form a two-component signal transduction system that may help bacteria sense available substrates and respond to the changing environments (79). There are cases when sulfatases themselves cluster together, for example, four or six copies in a row. A complete pathway for assimilatory sulfate reduction is also present in the genome and the cells may utilize the cleaved sulfate group for biosynthesis of reduced sulfur compounds.

A comparative study of GHs and sulfatases in selected genomes of the *Kiritimatiellales* order revealed that not all genomes harbor enzymes involved in degradation pathways of agar, ɩ-carrageenan, and fucoidan, and some bacteria don’t have any GHs or sulfatases at all (Fig. 2; Table S5). However, certain genomes in the *Pontiella* genus show a relatively larger component of GHs and sulfatases. This indicates that these bacteria may adopt the lifestyle of utilizing macroalgal polysaccharides like agar, carrageenan, and fucoidan as carbon and energy sources, while other clades in the *Kiritimatiellales* order may specialize on other substrates available in their living environments. Some genomes in the *Pontiellaceae* family do not have a high number of GHs or sulfatases either. This may indicate that these carbohydrate-related genes could be laterally transferred into the *Pontiella* genus, but some were lost during evolution while living in environments where other available substrates were preferred. For example, such phenomenon was reported that the lateral gene transfer of porphyranases was from the marine *Bacteroidota*, *Zobellia galactanivorans*, to the human gut bacterium *Bacteroides plebeius* (80). Another possibility would be that these metagenome-assembled genomes (MAGs) were incomplete, and the GHs or sulfatases investigated were not easy to capture.

### Nitrogen fixation

**Fig 6:**
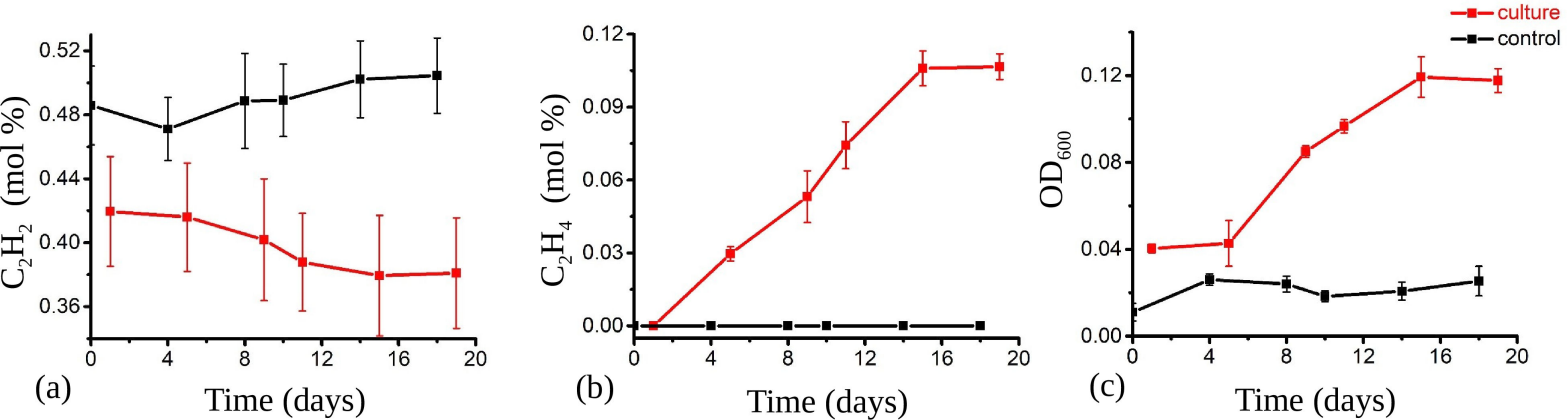
Nitrogenase activity of strain NLcol2^T^ detected by acetylene reduction assay. Acetylene (**a**), ethylene (**b**) percentage, and OD_600_ (**c**) change with time in liquid culture with D-galactose as substrate and nitrogen gas as the sole N source. Cultures with bacteria are labeled in red, and media without inoculum (control) are labeled in black. The decrease of acetylene and the increase of ethylene indicated active nitrogen fixation during bacterial growth.

We further tested nitrogen-fixing ability in live cultures of strain NLcol2^T^. The strain was able to grow on nitrogen gas as the sole N source in a nitrogen-free medium with D-galactose as the carbon source. No growth was observed when nitrogen was replaced by helium in the headspace. Bacterial growth was also supported by ammonium but not nitrate (Fig. S5), and neither assimilatory nor dissimilatory nitrate reductase was present in the genome. Nitrogenase activity was detected by acetylene reduction assay. The production of ethylene from acetylene during bacterial growth on nitrogen gas as the sole nitrogen source showed that the cultures expressed active nitrogenases and could fix nitrogen gas into bioavailable forms to support their growth (Fig. 6). This nitrogen-fixing ability may give them the advantage to survive in nitrogen-limiting environments.

Mo-dependent nitrogenase is the most common and widely studied enzyme that performs nitrogen fixation. It contains two components: an Fe protein as the reductase (*nifH*) collecting and transferring electrons and a MoFe protein (*nifDK*) binding dinitrogen (N_2_) and converting it to ammonia (NH_3_; 81). Genes encoding both nitrogenase iron protein (*nifH*, PF00142) and nitrogenase molybdenum-iron protein alpha and beta subunits (*nifDK*, PF00148) are present in the genome, which together form a complete pathway of nitrogen fixation. No alternative vanadium-iron nitrogenase or iron-only nitrogenase was found. In addition to *nifHDK*, both *nifB* and *nifE* involved in the biosynthesis of nitrogenase MoFe cofactor are present in the genome. Two genes coding for nitrogen regulatory protein PII were present, which are important for the regulation of nitrogen fixation in response to nitrogen source availability (82). The rop-like protein is uncharacterized but often found in nitrogen fixation operons and may play a role in regulation (83). There are various other *nif* genes present in other parts of the genome including *nifA*, *M*, *S*, *U*, and *V*, which together may help regulate the function of nitrogenase (Table S6).

Nitrogenases are highly oxygen-sensitive, but even though there are diverse anaerobes in the PVC superphylum, only a few studies demonstrated nitrogen fixation in this superphylum (84–87), and no reports in the *Kiritimatiellota* phylum. Moreover, we have little knowledge as to where *nif* genes were acquired by the nitrogen-fixing members in the PVC superphylum. We found five genomes in this phylum housing a *nifH* gene. Three were from *P. desulfatans*, *P. sulfatireligans*, and isolate S94, and two were from the marine sediments at the hydrothermal vent of South Mid-Atlantic Ridge (SZUA-380 and SZUA-494). All *nifH* genes in this clade were classified as cluster III, which is dominated by distantly related obligate anaerobes (88). All six *nifH* genes from the *Kiritimatiellota* phylum form a monophyletic clade with a bootstrap value of 89 (Fig. S6). They also cluster together with sequences from *Chlorobi*, *Bacteroidota*, and *Delataproteobacteria* (mainly the *Desulfovibrio* genus), *Spirochaetes*, and some *Verrucomicrobia* to form a monophyletic clade with a bootstrap value of 85. This suggests that there could be lateral gene transfer between the *Kiritimatiellota* phylum and other phyla in this clade, but some bacteria in the *Kiritimatiellota* phylum may have lost *nif* genes during evolution. Nitrogen fixation genes in a methanotrophic verrucomicrobial isolate *Methylacidiphilum fumariolicum* strain SolV resemble those from the *Gammaproteobacteria*, which supports their acquisition of *nif* genes through lateral gene transfer (84).

### Conclusion

In this study, we reported a novel anaerobic bacterial strain NLcol2^T^ isolated from microbial mats in marine sediments as the representative of a novel species in the *Pontiella* genus, which is the fifth cultivated strain in the *Kiritimatiellota* phylum. It represents the first strain to utilize agar as a substrate with nitrogen-fixing ability in the *Kiritimatiellota* phylum. An extensive list of CAZymes and sulfatases shows its potential to degrade diverse macroalgae-derived sulfated polysaccharides in marine environments.

### Description of *Pontiella agarivorans* sp. nov.

*Pontiella agarivorans* (a.ga.ri.vo'rans. N.L. neut. n. *agarum* agar, algal polysaccharide; L. pres. part. adj. *vorans* devouring, consuming; N.L. part. adj. *agarivorans* agar-devouring).

Cells are Gram-negative, anaerobic, nonmotile cocci with a diameter of 1 µm. No spore formation was observed. Cells divide by binary fission. Colonies on agar plates are milky or ivory, circular, and smooth. Growth occurs at 10–37°C (optimum 31°C), with NaCl concentration between 10 and 60 g/L (optimum 25–30 g/L) and with pH 6.0–9.0 (optimum pH 8.0) when D-galactose was utilized as the substrate. The following substrates support growth: D-glucose, D-galactose, D-fructose, D-mannose, D-mannitol, D-xylose, D-cellobiose, lactose, sucrose, maltose, xylan, agarose, agar, ɩ-carrageenan, and fucoidan. The following compounds do not support growth under laboratory conditions: L-fucose, L-rhamnose, D-arabinose, meso-inositol, starch, cellulose, or alginate. The nongaseous fermentation products from D-galactose are succinate, acetate, and malate (traces). Both ammonium and nitrogen gas can be utilized as nitrogen sources, but nitrate and nitrite were not utilized. Major cellular fatty acids are C18:0, *i-*C12:0, and *i-*C18:0.

The type strain NLcol2^T^ (= DSM 113125^T^ = MCCC 1K08672^T^), was isolated from microbial mats on the surface of marine sediments offshore Santa Barbara, CA, USA. The genome of the type strain is 4.4 Mbp in size, and DNA G + C content is 52.4 mol%. The GenBank accession number for the full-length 16S rRNA gene sequence of strain NLcol2^T^ is OQ749723. The genome of strain NLcol2^T^ has been deposited as the Whole Genome Shotgun project at DDBJ/ENA/GenBank under the accession number JARVCO000000000. The version described in this paper is version JARVCO010000000.

## Data Availability

Strain NLcol2^T^ has been deposited at Leibniz-Institut DSMZ (= DSM 113125^T^) and Marine Culture Collection of China (= MCCC 1K08672^T^). The GenBank accession number for the full-length 16S rRNA gene sequence of strain NLcol2^T^ is OQ749723. The genome of strain NLcol2^T^ was has been deposited as the Whole Genome Shotgun project at DDBJ/ENA/GenBankNCBI under the accession number JARVCO000000000. The version described in this paper is version JARVCO010000000.

## SUPPLEMENTAL MATERIAL

Supplemental Figures S1-S6 aem.00914-23-s0001.pdf Supplemental figures.

Supplemental Table S1 aem.00914-23-s0002.xlsx FAMEs analyses.

Supplemental Table S2 aem.00914-23-s0003.xlsx CAZymes.

Supplemental Table S3 aem.00914-23-s0004.xlsx Sulfatases.

Supplemental Table S4 aem.00914-23-s0005.xlsx Potential genes involved in macroalgal polysaccharide degradation.

Supplemental Table S5 aem.00914-23-s0006.xlsx Comparative genomic data for heatmap.

Supplemental Table S6 aem.00914-23-s0007.xlsx nif genes.

ASM does not own the copyrights to Supplemental Material that may be linked to, or accessed through, an article. The authors have granted ASM a non-exclusive, world-wide license to publish the Supplemental Material files. Please contact the corresponding author directly for reuse.
