# Supplementary material for: Pontiella agarivorans sp. nov., a novel marine anaerobic bacterium capable of degrading macroalgal polysaccharides and fixing nitrogen
Source: Appl Environ Microbiol. 2024 Jan 24;90(2):e00914-23. doi: 10.1128/aem.00914-23 (PMC10880615; doi:10.1128/aem.00914-23)
Supplement: Supplemental Figures S1-S6 — Supplemental figures. [file aem.00914-23-s0001.pdf]

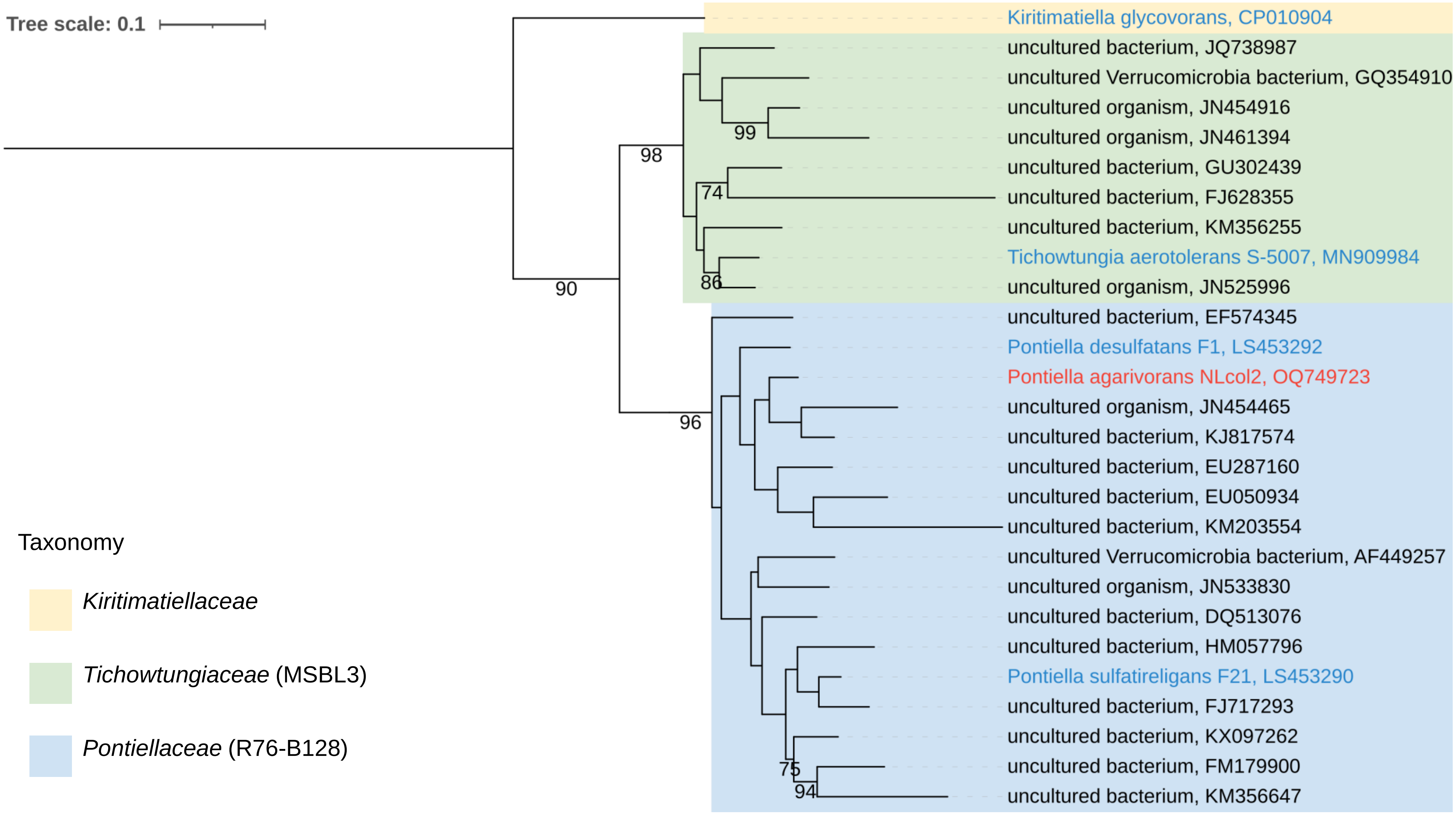

Figure S1. Maximum-likelihood phylogenomic tree of 16S rRNA genes of the *Kiritimatiellales* order. Strain NLcol2 is labeled in red and other cultivated strains are labeled in blue. The tree was rooted with two *Verrucomicrobia* sequences (not shown) and pruned with 27 selected representative sequences. Bootstrap values over 70 are shown on the nodes.

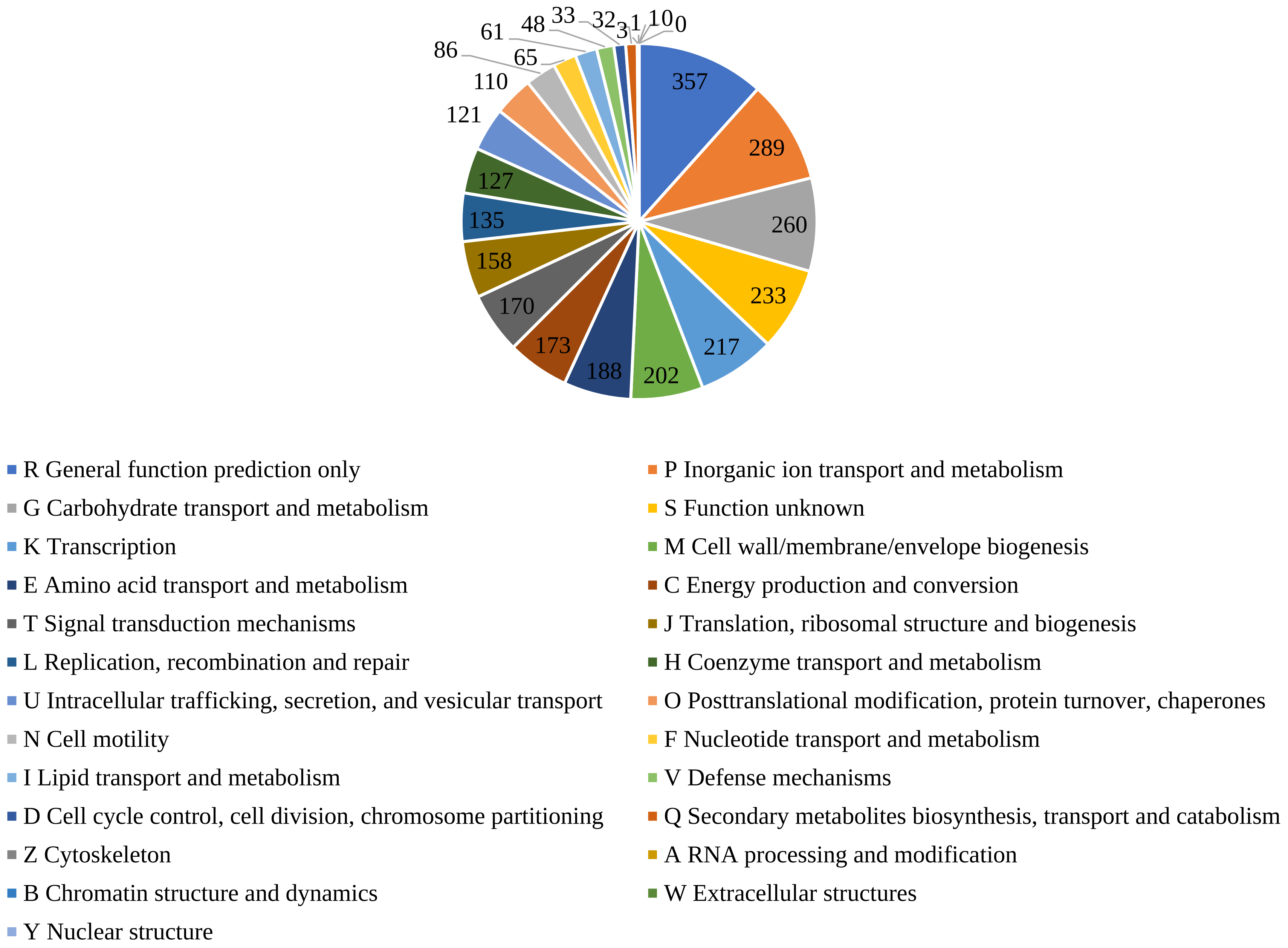

Figure S2. Pie chart showing number of genes assigned in COG (Cluster of Orthologous Groups) functional categories.

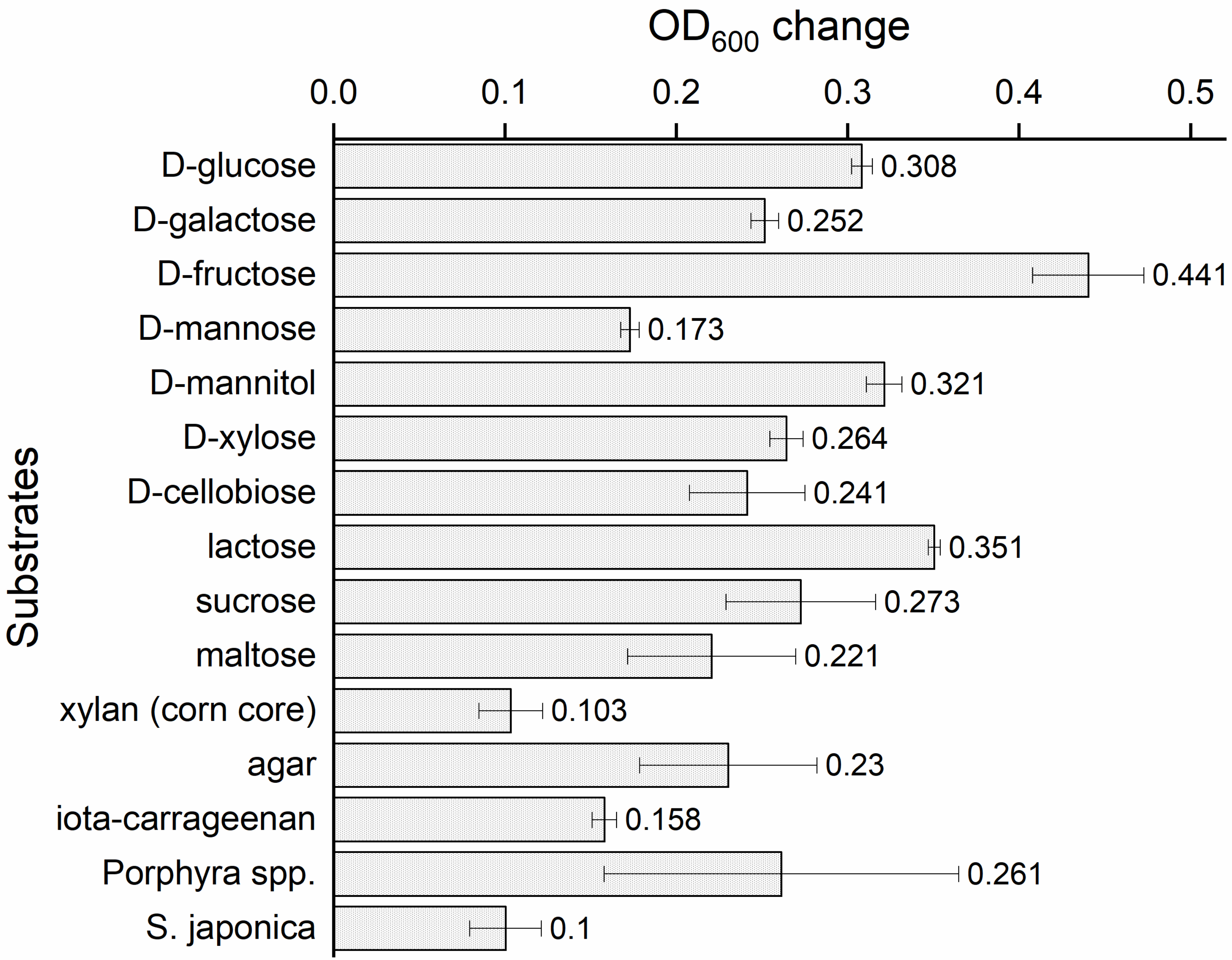

Figure S3. Bacterial growth of strain NLcol2^T observed by the change of optical density at 600nm (OD_{600}) between the start and end timepoints of the incubations when supplied with various substrates. The list of substrates only includes those that can support the growth of strain NLcol2^T. The average values of OD change from triplicates were labeled on the side of the bars.

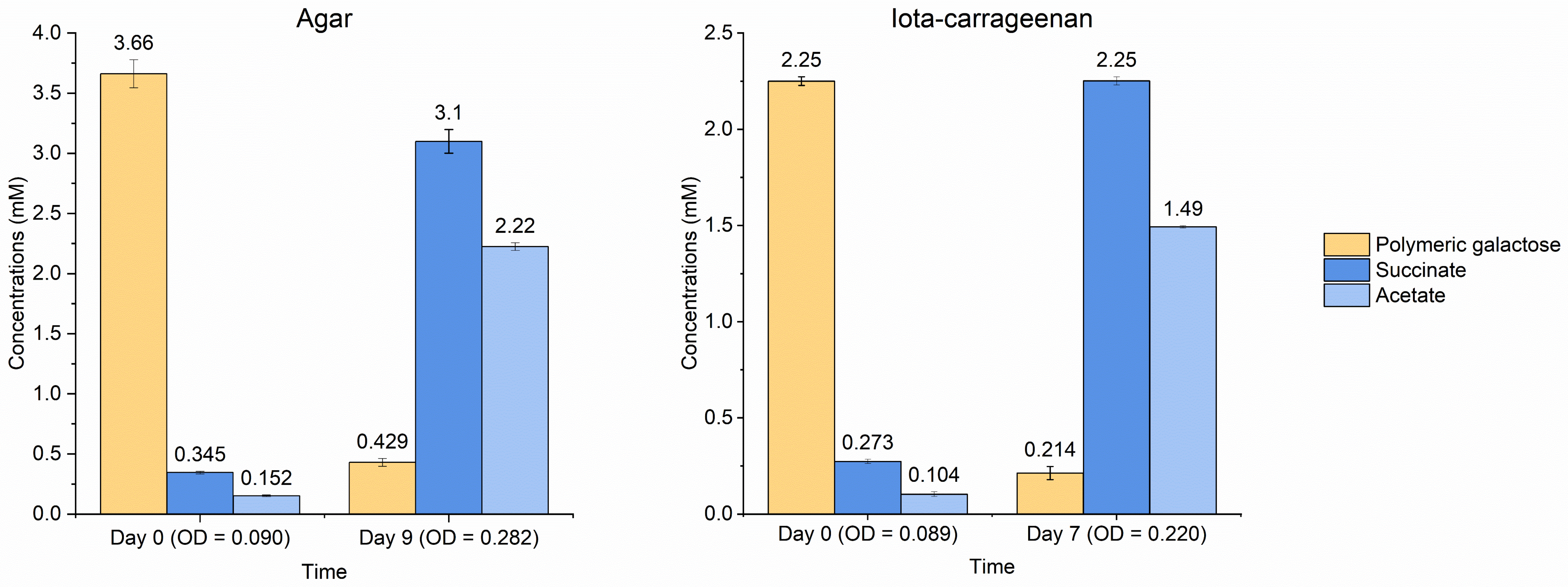

Figure S4. Agar and iota-carrageenan degradation by strain NLcol2^{T}. Agar and iota-carrageenan concentrations were quantified as polymeric galactose after acid hydrolysis. Metabolites of succinate and acetate were measured as well. Bacterial growth was measured by optical density at 600nm (OD_{600}). The average values of concentrations from triplicates were labeled on the top of bars. Agar and iota-carrageenan concentrations decreased while fermentation products of succinate and acetate were produced during the growth of cultures.

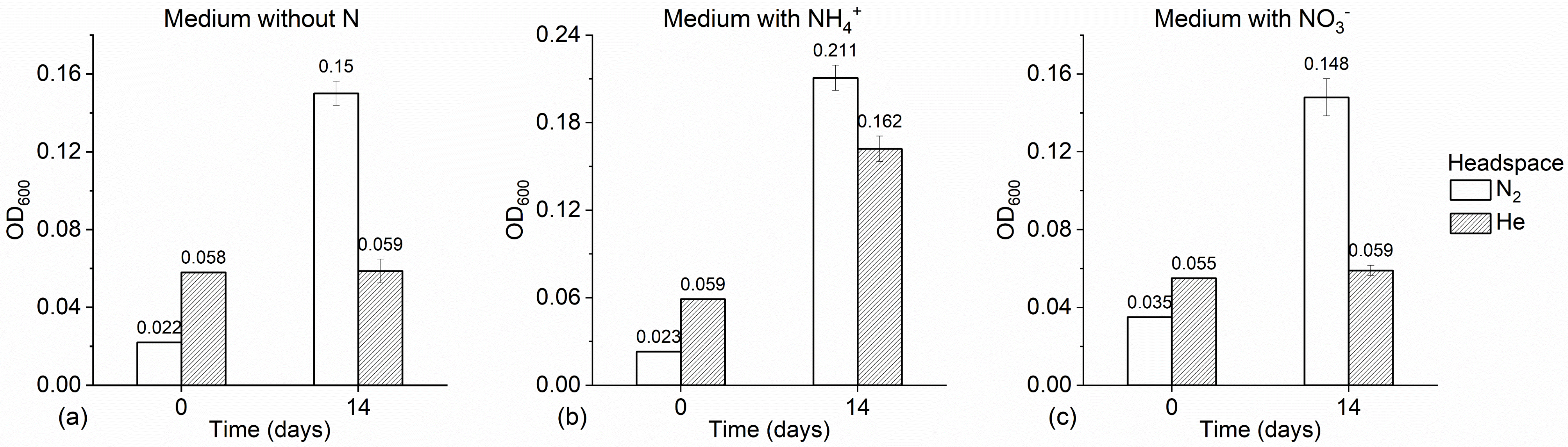

Figure S5. Comparisons of strain NLcol2^T cultures growing in media with (a) no N, (b) ammonium or (c) nitrate supplemented. Headspace gases are nitrogen gas (white bar) or helium (grey bar). OD_{600} was measured at the beginning (day 0) and at the end (day 14) of incubation. The OD values were labeled on the top of bars. Bacterial growth was supported by nitrogen gas, ammonium, but not nitrate.

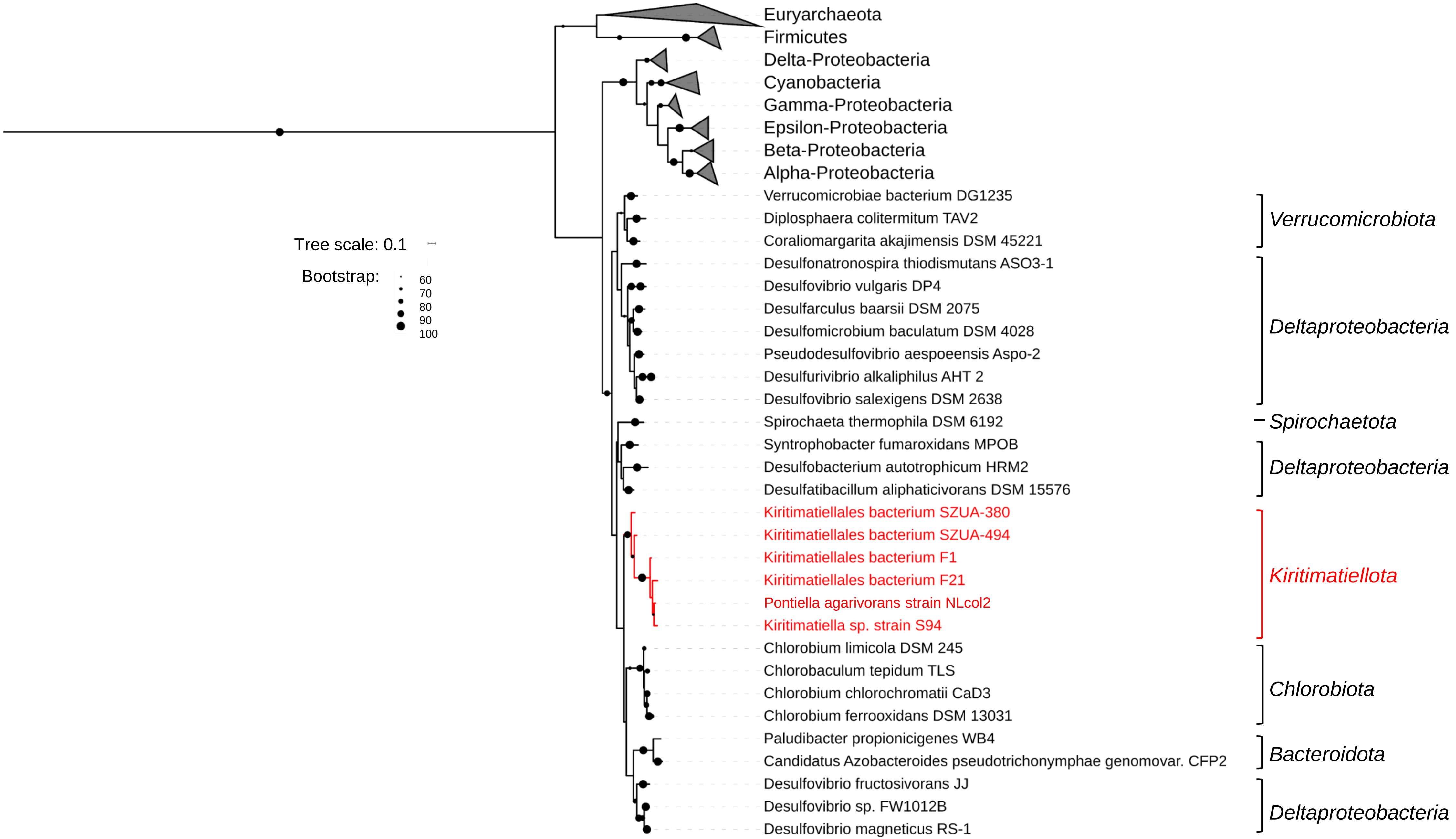

Figure S6. Phylogenetic placement of *nifH* genes in the *Kiritimatiellota* phylum among 879 *niH* genes from cultivated diazotrophs on a maximum-likelihood tree. Two light-independent protochlorophyllide reductases were included as outgroups (not shown) and the tree was rooted there. The tree was pruned with 48 representatives of different phyla. Bootstrap values over 60 are shown in dots on the nodes.
